# Circular RNA NEK6 contributes to the development of non-small-cell lung cancer by competitively binding with miR-382-5p to elevate BCAS2 expression at post-transcriptional level

**DOI:** 10.1186/s12890-021-01617-0

**Published:** 2021-10-18

**Authors:** Fei Cao, Xiaoxia Wu, Yongfeng Shan, Binbin Zhang, Haonan Wang, Hui Liu, Hao Yu

**Affiliations:** Department of Oncology, Wuxi Fifth People’s Hospital, No. 1215 Guangrui Road, Wuxi, 214016 China

**Keywords:** circ_NEK6, miR-382-5p, BCAS2, Non-small-cell lung cancer

## Abstract

**Background:**

Non-small-cell lung cancer (NSCLC) is the commonest type of lung cancer, which is one of most deadly cancers that possess high morbidity and mortality all over the world. The function of circular RNA NIMA related kinase 6 (circ_NEK6) in NSCLC is still unknown. Therefore, circ_NEK6 is worth studying in detail.

**Methods:**

RT-qPCR and western blot assays were employed to detect gene expression. Colony formation, EdU, JC-1, flow cytometry, and Transwell assays were implemented to explore the function of circ_NEK6 on biological activities of NSCLC cells. Mechanism experiments were conducted to unveil the relationship among molecules.

**Results:**

Circ_NEK6 expression was highly expressed in NSCLC tissues and cells. Functionally, the silencing of circ_NEK6 could effectively suppress NSCLC cell proliferation, migration and invasion. Circ_NEK6 sequestered miR-382-5p to fortify the expression of breast carcinoma amplified sequence 2 (BCAS2) in NSCLC. Besides, BCAS2 had tumor-promoting function in NSCLC. Furthermore, the effects of down-regulated circ_NEK6 on the malignant behaviors of NSCLC cells were totally recovered by miR-382-5p inhibition or BCAS2 overexpression.

**Conclusions:**

Circ_NEK6 served as a competing endogenous RNA (ceRNA) of BCAS2 by absorbing miR-382-5p, which may be treated as a novel promising target for the treatment of NSCLC.

**Supplementary Information:**

The online version contains supplementary material available at 10.1186/s12890-021-01617-0.

## Background

Lung cancer is regarded as one of the most deadly cancers with high incidence [1]. Nowadays, lung cancer is not considered as a single disease any more, which can be divided into different subtypes [2]. Among them, non-small-cell lung cancer (NSCLC) is identified as the most frequent subtype of lung cancer with high morbidity and mortality rate around the world [3, 4]. Currently, there are multiple therapy options for NSCLC patients, such as surgery, radiation therapy, chemotherapy, and targeted therapy [5]. Although diagnosis and treatments are continuously improved and optimized, the survival rate of patients with NSCLC is still disappointing. Hence, discovering novel and effective therapies is still urgent for NSCLC [3].

Circular RNAs (circRNAs), as a sub-class of non-coding RNA family, are featured with a covalently closed loop with neither a 5’ cap nor a 3’ polyadenylated tail [6, 7]. In the past several decades, growing circRNAs have been reported to play a significant role in various tumors, therefore becoming novel and promising therapeutic targets [8]. For example, circRNA_100290 exerts its cancer-promoting function in oral cancer [9]. Furthermore, circRNA_100876 is closely related to the carcinogenesis of NSCLC and has prognostic value for NSCLC patients [10]. In recent years, circRNA_NEK6 has already been reported as a carcinogenic factor in thyroid cancer [11]. However, the role of circ_NEK6 in NSCLC has not been reported.

MicroRNAs (miRNAs) are small noncoding RNAs that regulate gene expression via recognition of cognate sequences and interference of transcriptional, translational or epigenetic processes [12]. It has been found that lncRNA SNHG14 regulates SPIN1 expression to accelerate the tumor progression of NSCLC by sponging miR-382-5p [13]. However, whether there existed a binding relationship between circ_NEK6 and miR-382-5p in NSCLC was still unknown. Also, as miRNAs can exert functions via modulating the downstream protein-coding mRNA targets through RISCs. It was speculated by us that there may exist some potential targets of miR-382-5p that could mediate the function of circ_NEK6 in NSCLC.

In this study, we aimed to confirm the function and molecular mechanism of circ_NEK6 in NSCLC.

## Methods

### Sample collection

The NSCLC tissues and paired non-tumor tissues (n = 70) were acquired from our hospital with the approval of the Ethics Committee of our hospital. The written informed consents were obtained from all these 70 NSCLC patients who received no pre-operative treatment. Samples were all kept at − 80 °C after sharp-frozen in liquid nitrogen.

### Cell culture

Human normal bronchial epithelioid cell line (16HBE) and 4 human NSCLC cell lines (NCI-H23, PC-9, A-549 and NCI-H1869) were utilized in our research. All cell lines were bought from ATCC Company (Manassas, VA). RPMI-1640 medium was supplemented with 10% FBS and 1% P/S for cell cultivation at 37 °C with 5% CO_2_.

### Total RNA extraction and quantitative real-time polymerase chain reaction (RT-qPCR)

Trizol reagent (Invitrogen) was utilized to extract total RNA. Then, cDNA synthesis was accomplished by PrimeScript™ RT reagent kit in line with user guide. After that, qRT-PCR was conducted with SYBR Premix Ex Taq II (Takara), followed by gene expression examination by 2^−ΔΔCt^ method. GAPDH or U6 served as the control.

### Plasmid transfection

The shRNAs and NC-shRNAs were devised and constructed by GenePharma (Shanghai, China) and then utilized to transfect NCI-H23 and PC-9 cells so as to knock down circ_NEK6 and BCAS2. As for overexpression, the full-length cDNA sequence of BCAS2 was inserted into pcDNA3.1 vector (Invitrogen). Moreover, miR-382-5p mimics/inhibitor and NC mimics/inhibitor were obtained from GenePharma. Lipofectamine 2000 (Invitrogen) was utilized in cell transfection for 48 h.

### Fluorescence in situ hybridization (FISH)

First of all, 4% formaldehyde was utilized to fix NCI-H23 and PC-9 cells for 18 h which were then rinsed by PBS. After dehydration, cells were cultivated with circ_NEK6-FISH probe (Ribobio, Guangzhou, China). Three hours after hybridization, DAPI was adopted to stain cell nuclei. In the end, fluorescence microscope was utilized to observe and analyze the stained cells (Olympus Corp., Tokyo, Japan).

### Subcellular fraction assay

PARIS™ Kit (Invitrogen) was utilized to conduct this assay in NCI-H23 and PC-9 cells. After centrifugation, cells were subjected to cell disruption buffer. Finally, circ_NEK6 in cytoplasm/nuclei was examined by RT-qPCR.

### Colony formation assay

NCI-H23 and PC-9 cells (800 cells per well) were cultivated in 96-well plates for fortnight. After 14 days, the culture medium was discarded and the cells were washed with PBS for two times. Cells were then fixed with 4% formaldehyde for 15 min and stained by 0.5% crystal violet for 10 min at room temperature. In the end, the quantity of colonies (with over 50 cells) was counted manually.

### 5-Ethynyl-2′-deoxyuridine (EdU) assay

EdU cell proliferation kit (Ribo, Guangzhou, China) was utilized to measure the proliferative capability of NCI-H23 and PC-9 cells. After being treated with EdU for 3 h, cells were rinsed by PBS for 3 times. Then DAPI was applied to stain nuclei at room temperature (RT) in the darkroom. Finally, cells were observed under a light microscope.

### Transwell assay

NCI-H23 and PC-9 cells (2 × 10^4^) in serum-free culture medium were put into the upper chamber. As for invasion assay, we coated Matrigel on the upper chamber before experiments. Then, complete culture medium was added to the lower chamber. Twenty-four hours later, cells in the upper chamber were removed with caution by a cotton swab and then subjected to fixation with 4% formaldehyde for 15 min and stained by 0.5% crystal violet for 10 min in succession. Finally, cell numbers was computed by utilizing light microscope.

### Flow cytometry assay

After transfected for 48 h, NCI-H23 and PC-9 cells were gathered to the 6-well plates with 3 × 10^3^ cells per well. Next, Annexin V/PI Kit (BD Biosciences, San Jose, CA) were adopted for double-staining (15 min). Ultimately, the apoptosis of NCI-H23 and PC-9 cells was examined via BD Biosciences FACSCalibur flow cytometer.

### JC-1 assay

The transfected NCI-H23 and PC-9 cells were cultured in 96-well black microplate overnight. Then, after the cells were centrifuged, the culture medium was removed. After that, cells were stained with JC-1. Half an hour later, cells were counted through utilizing fluorescence microscope.

### Western blot

The transfected cells were collected and lysed in RIPA lysis buffer. BCA kit was employed to test the concentration of total protein. Thereafter, proteins were isolated by 12% SDS-PAGE and then shifted to PVDF membranes. After that, membranes were blocked with 5% nonfat milk and then cultivated with primary and secondary antibodies in succession. The primary antibodies specific to GAPDH and MMP2, MMP9, as well as HRP-tagged secondary antibodies, were acquired from Abcam (Cambridge, MA). Then, ECL system was adopted to evaluate the band signals in line with user guide (Amersham Pharmacia, Piscataway, NJ).

### RNase R and Actinomycin D (ActD) treatment

With regard to RNase R treatment, the total RNA was cultivated with or without RNase R (Epicentre Technologies) for half an hour at RT. As for ActD treatment, cells were supplemented to the ActD (Sigma‐Aldrich) or control DMSO (Sigma‐Aldrich) for indicated times (0, 4, 8, 12 h). Circ_NEK6 and the linear NEK6 in indicated cells were analyzed via RT-qPCR.

### RNA Immunoprecipitation (RIP) assay

The cell lysates were gathered utilizing RIP lysis buffer and then subjected to incubation with anti-AGO2 antibody conjugated in magnetic beads. Groups with anti-IgG antibody served as the negative control. After purification, RNAs in immunoprecipitation were detected by RT-qPCR.

### RNA pull down assay

RNA pull down assay was conducted with Pierce Magnetic RNA–Protein Pull-Down Kit which was obtained from Thermo Fisher Scientific. In short, cell lysates were mixed with biotin-labeled RNA probes of circ_NEK6 and magnetic beads. In the end, RT-qPCR was performed for analyzing RNAs in pulled down compounds.

### Luciferase reporter assay

Full-length sequences of circ_NEK6 and BCAS2 3’UTR with wild-type and mutant miR-382-5p binding sites were sub-cloned into pmirGLO luciferase vectors (Promega, Madison, WI) to acquire circ_NEK6-WT/MUT and BCAS2-WT/MUT. After that, NCI-H23 and PC-9 cells were subjected to co-transfection with above pmirGLO vectors and miR-382-5p mimics or NC mimics. 48 h later, the luciferase intensity was evaluated via Luciferase Reporter Assay System (Promega).

### Statistical analyses

Each assay was repeated at least three times. All results were analyzed by PRISM 6 (GraphPad, San Diego, CA), and then displayed as the mean ± SD. In addition, the statistical analysis was progressed through student’s *t*-test or one‐way ANOVA. P < 0.05 was considered as significant.

## Results

### Circ_NEK6 plays a carcinogenic role in NSCLC

**Fig. 1:**
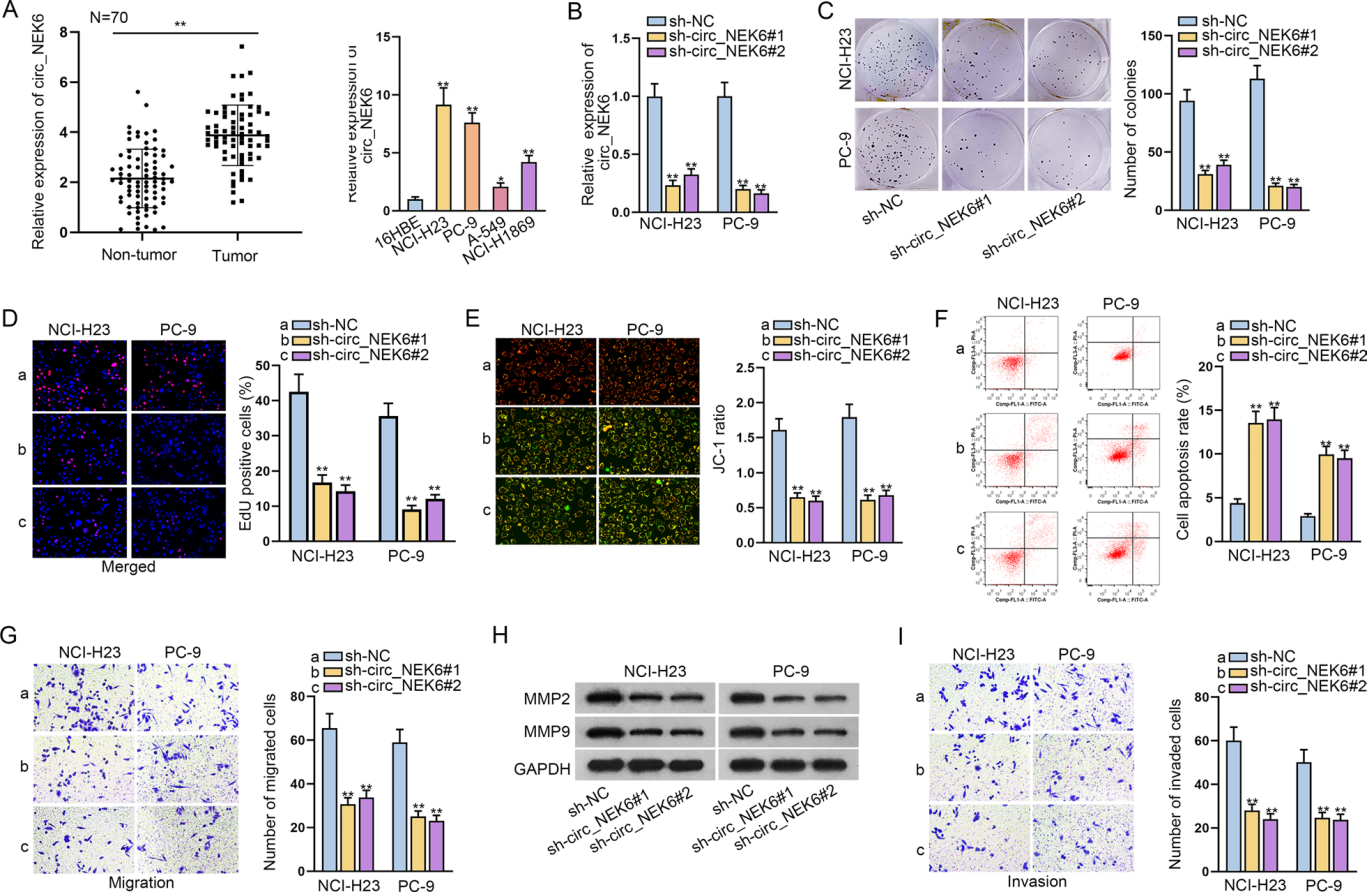
Circ_NEK6 plays a carcinogenic role in NSCLC. **A** Relative expression of circ_NEK6 in 70 pairs of NSCLC samples was examined, and circ_NEK6 expression was detected by RT-qPCR in normal 16HBE cells and NSCLC cells (including NCI-H23, PC-9, A-549 and NCI-H1869). **B** Interference efficiency of shRNAs targeting circ_NEK6 was assessed via RT-qPCR assay. **C**, **D** Colony formation and EdU assays were conducted to uncovered the effect of circ_NEK6 down-regulation on cell proliferation. **E**, **F** The apoptosis abilities of NSCLC cells were assessed by JC-1 assay and flow cytometry analysis. **G** Transwell assay was performed to detect the migration of NSCLC cells. **H** Western blot was conducted to analyze the protein level of MMP2 and MMP9 proteins in cells with silenced circ_NEK6 or not. **I** The capacity of cell invasion was detected by Transwell assay. All results were displayed as the mean ± SD. *P < 0.05, **P < 0.01

In the past few years, the role of circRNAs in different cancers has been studied and discussed by the experts in medical and biology fields. However, the expression pattern and molecular mechanism of circ_NEK6 in the NSCLC remain to be explored. We observed that circ_NEK6 expression was elevated in NSCLC tumors compared to paired non-tumor tissues. Then, we also adopted RT-qPCR to examine circ_NEK6 expression in normal 16HBE cells and NSCLC cells (including NCI-H23, PC-9, A-549 and NCI-H1869) (Fig. 1A). Consistently, circ_NEK6 expression was confirmed to be higher in NSCLC cells lines than in 16HBE cells. Due to the higher expression of circ_NEK6 in NCI-H2 and PC-9 cell lines than that in the other ones (A-549 and NCI-H1869), we took NCI-H23 and PC-9 cells as the subjects of the follow-up experiments. To carry out loss-of-function assays, we firstly down-regulated circ_NEK6 expression in NCI-H23 and PC-9 cells by the transfection with sh-circ_NEK6#1 and sh-circ_NEK6#2, as validated in Fig. 1B. After that, colony formation and EdU assays were conducted by us and the relevant results demonstrated that down-regulated circ_NEK6 effectively cut down the number of colonies and decreased the percentage of EdU positive cells in both NCI-H23 and PC-9 cells (Fig. 1C, D). On the contrary, the results of flow cytometry analysis and JC-1 assay clearly demonstrated that the knockdown of circ_NEK6 significantly enhanced NSCLC cell apoptosis (Fig. 1E, F). Additionally, we also measured the migration and invasion of NCI-H23 and PC-9 cells by utilizing Transwell assays. It was obviously shown that the number of migrated cells was sharply decreased due to circ_NEK6 inhibition (Fig. 1G). Besides, the declined protein levels of MMP2/MMP9 under circ_NEK6 down-regulation further verified the suppressed cell migration by down-regulating circ_NEK6 (Fig. 1H). Likewise, the invasion ability was also observed to be effectively hindered after inhibiting circ_NEK6 (Fig. 1I). Depending on the results above, we concluded that down-regulating circ_NEK6 in NSCLC cells could hinder cell proliferation, migration and invasion while enhancing cell apoptosis.

### MiR-382-5p is the downstream molecule of circ_NEK6 in NSCLC

**Fig. 2:**
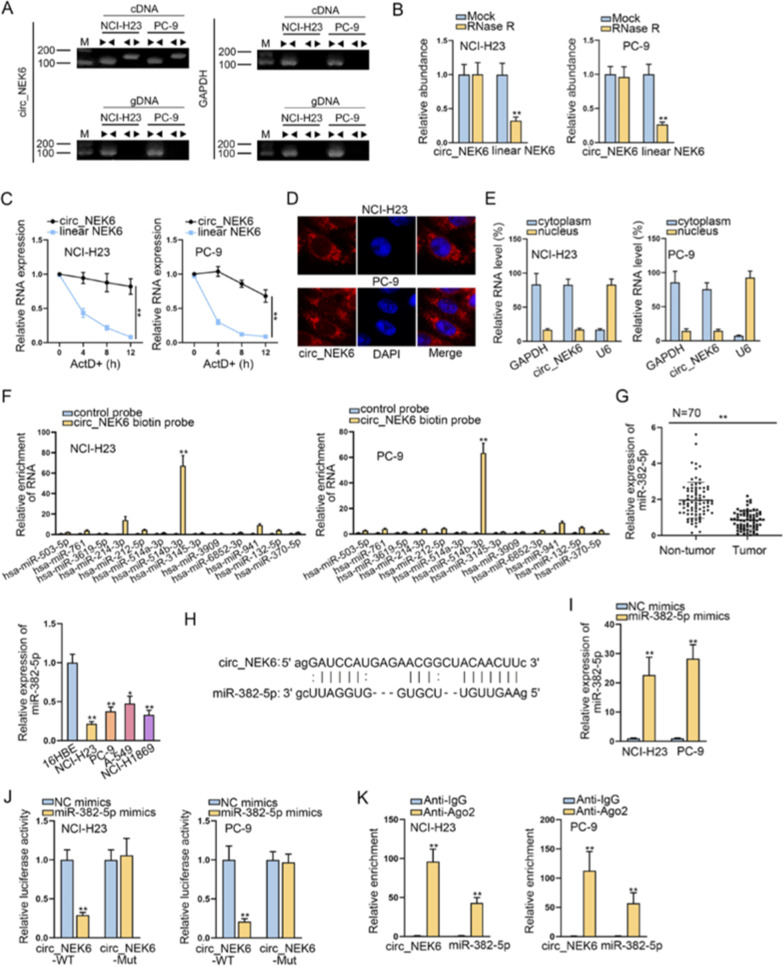
MiR-382-5p is the downstream of circ_NEK6 in NSCLC. **A** Agarose gel electrophoresis analysis was used to detect the circular feature of circ_NEK6. **B** RT-qPCR assay was used to assess circ_NEK6 expression in cells treated with RNase R. **C** The expression of linear NEK6 and circ_NEK6 under ActD treatment for indicated time was detected by RT-qPCR. **D**, **E**. FISH and subcellular separation assays were used to confirm the subcellular location of circ_NEK6. **F** RNA pull down assay was utilized to test the binding affinity of circ_NEK6 with 13 miRNAs. **G** MiR-382-5p expression in NSCLC cells and 16HBE was detected by RT-qPCR assay, and relative expression of miR-382-5p in 70 pairs of NSCLC samples was examined. **H** The potential binding sites between circ_NEK6 and miR-382-5p were predicted by ENCORI. I. RT-qPCR assay was used to detect the overexpression efficiency of miR-382-5p mimics. J-K. Luciferase reporter and RNA pull down assays were used to confirm the association between circ_NEK6 and miR-382-5p. All results were displayed as the mean ± SD. **P < 0.01

Prior to the molecular mechanism exploration of circ_NEK6 in NSCLC, we aimed to unveil the circular characteristics of circ_NEK6 in the first place. As expected, agarose gel electrophoresis proved that circ_NEK6 could only be amplified in cDNA by divergent primer, not in gDNA or by convergent primer (Fig. 2A). Besides, it was manifested that after RNase R treatment, the abundance of linear NEK6 was obviously decreased whereas no obvious change was observed in that of circ_NEK6 (Fig. 2B). Consistently, when the cells were treated with ActD, the expression of linear NEK6 was greatly decreased compared with that of circ_NEK6 (Fig. 2C). Based on those data, we confirmed the circular structure circ_NEK6. Next, in order to determine the subcellular location of circ_NEK6, we applied FISH assay and nuclear separation assays. Consequently, we uncovered that circ_NEK6 was mainly distributed in the cytoplasm of NSCLC cells (Fig. 2D, E). Then, RNA pull down assay was conducted to determine the target miRNAs of circ_NEK6 in NCL-H23 and PC-9 cells. As shown by the result, it was presented that only miR-382-5p among 13 potential miRNAs (miR-370-5p, miR-3619-5p and miR941, etc.) was obviously enriched by circ_NEK6 biotin probe compared with that of control group (Fig. 2F). Thus, we chose miR-382-5p as the downstream miRNA of circ_NEK6 in NSCLC. As presented in Fig. 2G, the expression of miR-382-5p was apparently decreased in NSCLC cell lines compared with that in 16HBEcell line. Likewise, we also found that compared with the expression in non-tumor tissues, the miR-382-5p expression in 70 NSCLC tissues was obviously lower. Then, we used ENCORI (http://starbase.sysu.edu.cn/index.php) to predict the binding sites between circ_NEK6 and miR-382-5p (Fig. 2H). Further, to study the interaction between circ_NEK6 and miR-382-5p, we elevated miR-382-5p expression in NCI-H23 and PC-9 cells by transfecting with miR-382-5p mimics (Fig. 2I). Luciferase reporter assay was then carried out by us and the result elucidated that the group with circ_NEK6-WT presented decreased luciferase activity induced by the up-regulation of miR-382-5p, whereas no obvious change was observed in the group with circ_NEK6-Mut compared with the control group (Fig. 2J). Notably, after carrying out RIP assay, we unveiled that circ_NEK6 and miR-382-5p were both enriched by Anti-Ago2 but not by Anti-IgG (Fig. 2K), which explained that they could co-exist in RNA-induced silencing complexes (RISCs). In a word, circ_NEK6 and miR-382-5p interacted with each other in RISCs in NSCLC (Additional file 1: Fig. S1; Additional file 2; Additional file 3: Table S1; Additional file 4: Table S2; Additional file 5: Table S3; Additional file 6: Table S4).

### BCAS2 is the downstream target of miR-382-5p and exerts tumor-promoting functions in NSCLC

**Fig. 3:**
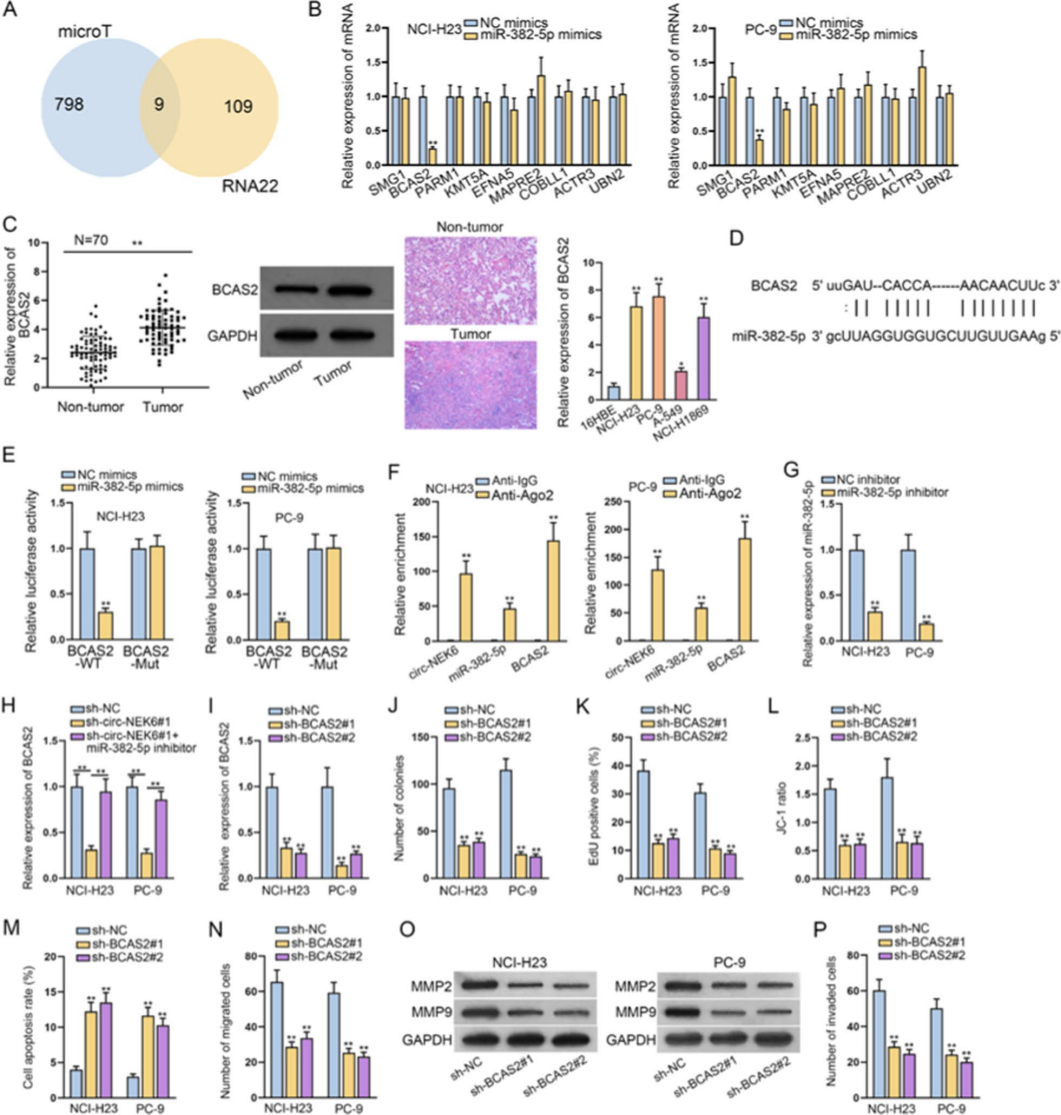
BCAS2 is the downstream target of miR-382-5p and exerts tumor-promoting functions in NSCLC. **A** MicroT and RNA22 were used to predict the potential targets for miR-382-5p. **B** RT-qPCR assay was carried out to analyze the effect of miR-382-5p on the expression of nine candidates in NSCLC cells. **C** BCAS2 expression in NSCLC cells compared to 16HBE cells and relative expression and protein levels of miR-382-5p in 70 pairs of NSCLC samples were examined by RT-qPCR and western blot assays. **D** ENCORI was used to predict the binding sites between miR-382-5p and BCAS2. **E** Luciferase reporter assay was used to confirm the interaction between miR-382-5p and BCAS2. **F** RIP assay was carried out to detect whether circ_NEK6, miR-382-5p and BCAS2 co-existed in RISCs. **G** The interference efficiency of miR-382-5p was detected. **I** RT-qPCR assay was adopted to detect the expression of BCAS2 under difference treatment conditions. **J**, **K** NSCLC cell proliferation was tested by colony formation and EdU assays upon BCAS2 silencing. **L**, **M** NSCLC cell apoptosis was evaluated by JC-1 assay and flow cytometry analysis upon BCAS2 silencing. **N** Transwell assay was conducted to assess NSCLC cell migration upon BCAS2 silencing. **O** The protein levels of MMP2 and MMP9 proteins in cells transfected with sh-BCAS2#1/2 were measured via western blot assay. **P** Transwell assay was used to detect NSCLC cell invasion upon BCAS2 silencing. All results were displayed as the mean ± SD. **P < 0.01

MiRNAs have been well-elaborated to exert functions via modulating the downstream protein-coding mRNA targets through RISCs [14]. Hence, we intended to figure out the potential target of miR-382-5p that mediated the function of circ_NEK6 in NSCLC. According to RNA22 and microT database, 9 mRNAs which might combine with miR-382-5p were sifted out (Fig. 3A). Thereafter, RT-qPCR was adopted to assess the expression of those mRNAs in NSCLC cells transfected with miR-382-5p mimics. As shown by the figures, it was uncovered that upon miR-382-5p up-regulation, the level of BCAS2 was markedly reduced in NSCLC cells while no noticeable changes were observed in that of other 8 mRNAs (Fig. 3B). Then, it was observed that both the protein and expression levels of BCAS2 in NSCLC tumors were increased. In addition, we found that BCAS2 expression in NSCLC cell lines presented higher levels compared with that in normal 16HBE cells (Fig. 3C). Moreover, the positive correlation of BCAS2 with circ_NEK6 and the negative association of BCAS2 with miR-382-5p in expression were disclosed in NSCLC specimens (Additional file 1: Figure S1A). Therefore, we chose BCAS2 for subsequent assays. Next, starBase (http://starbase.sysu.edu.cn/) was utilized to predict the potential binding sites between miR-382-5p and BCAS2 (Fig. 3D). As illustrated in Fig. 3E, only the luciferase activity of BCAS2-WT was cut down by miR-382-5p mimics. Furthermore, we found that circ_NEK6, miR-382-5p and BCAS2 were all prone to be enriched in Anti-Ago2 groups compared with Anti-IgG groups (Fig. 3F), confirming their coexistence in RISCs. After that, we detected the interference efficiency of miR-382-5p inhibitor and obtained a favorable result (Fig. 3G). Next, we observed that declined BCAS2 expression by sh-circ_NEK6#1 was effectively reversed after the co-transfection of miR-382-5p inhibitor (Fig. 3H). Meanwhile, we also evaluated the interference efficiency of sh-BCAS2 in NSCLC (Fig. 3I). After conducting several functional assays, we verified that silencing BCAS2 in NSCLC cells could result in impaired proliferation (Fig. 3J, K), elevated apoptosis (Fig. 3L, M), hindered migration (Fig. 3N, O), and suppressed invasion (Fig. 3P). In sum, BCAS2, which promoted the malignant behaviors of NSCLC, was the downstream target of miR-382-5p.

### Circ_NEK6 drives malignancy in NSCLC by modulating miR-382-5p/BCAS2 pathway

**Fig. 4:**
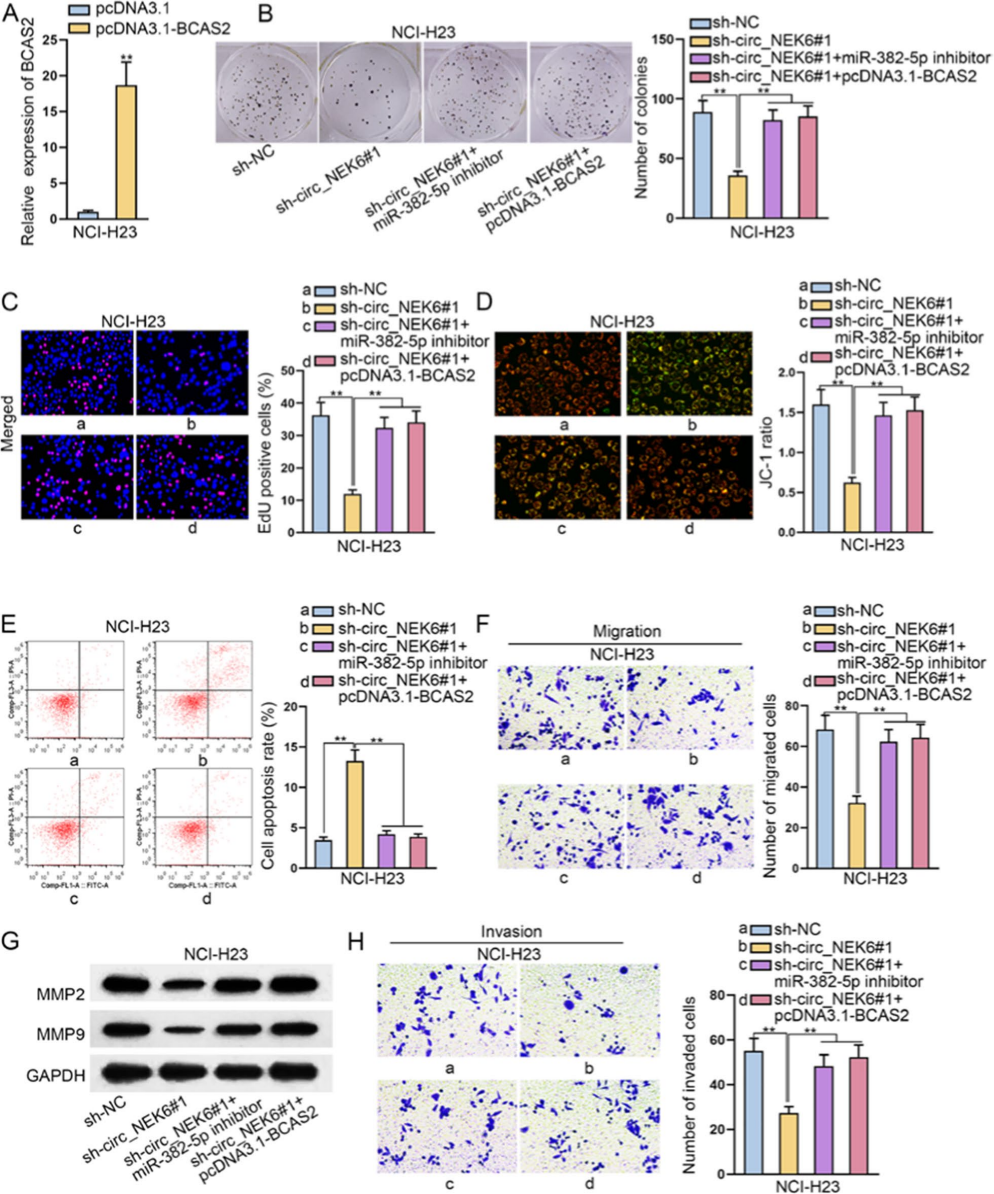
Circ_NEK6 drives NSCLC progression by modulating miR-382-5p/BCAS2 pathway. **A** Overexpression efficiency of BCAS2 was assessed via RT-qPCR assay. **B**, **C** Colony formation and EdU assays were performed to detect NSCLC cell proliferation under different conditions. **D**, **E** JC-1 assay and flow cytometry analysis were conducted to assess the apoptosis ability of NSCLC cells under different conditions. **F** Transwell assay was performed to detect the abilities of NSCLC cell migration under different conditions. **G** Levels of indicated proteins were tested via western blot assay under different conditions. **H** The capacity of cell invasion was observed by Transwell assay under different conditions. All results were displayed as the mean ± SD. **P < 0.01

Subsequently, we conducted several rescue assays to elucidate whether circ_NEK6 exerted functions on NSCLC through targeting miR-382-5p/BCAS2 axis. Firstly, we overexpressed BCAS2 with pcDNA3.1-BCAS2 in NCI-H23 cells and the result was detected by RT-qPCR analysis (Fig. 4A). Next, by conducting colony formation and EdU assays, it was revealed that cell proliferation was sharply reduced due to circ_NEK6 knockdown, but that result could be greatly reversed by the co-transfection of miR-382-5p inhibitor or pcDNA3.1-BCAS2 (Fig. 4B, C). In the meantime, we found that miR-382-5p silencing or BCAS2 up-regulation could neutralize the effect of inhibited circ_NEK6 on cell apoptosis (Fig. 4D, E). Additionally, we uncovered that the suppressed migratory capacity in circ_NEK6-deficient NSCLC cells could be greatly recovered by miR-382-5p down-regulation or BCAS2 up-regulation (Fig. 4F). Then, western blot assay was conducted by us and we observed that the changes of MMP2/MMP9 protein levels caused by sh-circ_NEK6#1 were greatly rescued by miR-382-5p inhibitor or pcDNA3.1/BCAS2 (Fig. 4G). As displayed in Fig. 4H, miR-382-5p down-regulation or BCAS2 up-regulation could recover the restrained invasive capacity of NSCLC cells caused by circ_NEK6 deficiency. All in all, circ_NEK6 played a carcinogenic role in NSCLC by modulating miR-382-5p/BCAS2 signaling.

## Discussion

Accumulating evidence has demonstrated the key functions of circRNAs in various malignancies [15, 16]. Also, the biological function and molecular mechanism of several circRNAs in NSCLC have been reported. On the one hand, some circRNAs exert the promoting functions in cancer. For instance, circ_0067934 overexpression results in accelerated cell proliferation and leads to a depressing survival rate among NSCLC patients [17]. CircP4HB enhances EMT and metastasis in NSCLC through sponging miR-133a-5p [18]. On the other hand, some circRNAs play a tumor-repressive role in cancer. For example, circ_PTPRA effectively suppresses EMT in NSCLC by sequestering miR-96-5p to up-regulate RASSF8 [19]. Circ_0001649 is decreased in NSCLC cells and inhibits cell growth and metastasis by regulating miR-331-3p and miR-338-5p [20]. In this study, the findings showed that silencing circ_NEK6 led to declined NSCLC cell proliferation, migration, invasion while facilitating cell apoptosis. In other words, circ_NEK6 served as an oncogene in NSCLC, which was consistent with its role in thyroid cancer [11].

Recent studies indicated that circRNAs can regulate protein-coding genes by sponging downstream miRNAs [21, 22]. Besides, such mechanisms have also been suggested to play key regulatory roles in diverse biological processes [23]. For instance, circRNA_100269 knockdown obviously suppresses gastric cancer cell growth via sponging miR-630 [24]. In the present study, we probed the relationship between circ_NEK6 and miR-382-5p. According to several current literatures, it have been reported miR-382-5p as a tumor-inhibitor in glioma [25] and osteosarcoma [26], and also indicated it as a tumor-promoter in breast cancer [27] and colorectal cancer [28]. In addition, we unveiled that cell proliferation suppressed by silencing circ_NEK6 was fully recovered by inhibiting miR-382-5p, suggesting a suppressive effect of miR-382-5p in NSCLC.

Furthermore, miRNAs can regulate its downstream target mRNAs to exert its biological functions [23, 29]. As an example, miRNA-221 targets TIMP2 to aggravate tumorigenesis in NSCLC [30]. In our study, BCAS2 was determined as the underlying target of miR-382-5p. Also, we unmasked the tumor-promoting function of BCAS2 in NSCLC, which was consistent with several previous studies in breast cancer [31], prostate cancer [32], and esophageal cancer [33]. Furthermore, our research recognized that BCAS2 mediated the contribution of circ_NEK6 to the malignant phenotypes in NSCLC cells. More importantly, we also proved the enhanced expression levels of circ_NEK6 and BCAS2 as well as the decreased expression of miR-382-5p in NSCLC specimens. Also, the positive relationship between the expression of circ_NEK6 and BCAS2 as well as the negative association between miR-382-5p and BCAS2 levels were confirmed in clinical NSCLC samples, which further strengthened the significance of circ_NEK6/miR-382-5p/BCAS2 axis in NSCLC development. What’s more, according to the correlation between BCAS2, circ_NEK6 and miR-382-5p expression patterns and clinical features of NSCLC patients, our conclusions were further proved.

### Limitations

The present study didn’t explain why and how circ_NEK6 was up-regulated in NSCLC samples and cell lines, which required for further exploration. In addition, the lack of in vivo data was another major limitation of our work and we will further the corresponding experiments in our future studies.

## Conclusions

In conclusion, our work elucidated that circ_NEK6 could facilitate the malignancy in NSCLC by sequestering miR-382-5p to augment BCAS2. Therefore, circ_NEK6/miR-382-5p/BCAS2 regulatory axis might provide new promising therapeutic targets for NSCLC.

## Supplementary Information

**Additional file 1: Fig. S1.** Detailed information of circ_NEK6/miR-382-5p/BCAS2 axis in NSCLC. A. Pearson’s correlation analysis indicated the association of BCAS2 expression with the level of circ_NEK6 or miR-382-5p in 70 NSCLC tissues. B. Relationship between genes and NSCLC stages was shown.

**Additional file 2**. Original results of western blot assays in the laboratory

**Additional file 3: Table 1.** Sequences of primers and reaction condition

**Additional file 4: Table 2.** Information on correlation between BCAS2 expression and clinical features of NSCLC patients

**Additional file 5: Table 3.** Information on correlation between circ_NEK6 expression and clinical features of NSCLC patients

**Additional file 6: Table 4.** Information on correlation between miR-382-5p expression and clinical features of NSCLC patients

## Data Availability

All data and materials obtained in this work have been provided within the manuscript and relevant additional files.

## Abbreviations

circ_NEK6: Circular RNA NIMA related kinase 6
NSCLC: Non-small cell lung cancer
BCAS2: Breast carcinoma amplified sequence 2
ceRNA: Competing endogenous RNA
qRT-PCR: Quantitative real-time polymerase chain reaction
FISH: Fluorescence in situ hybridization
EdU: 5-Ethynyl-2′-deoxyuridine
ActD: Actinomycin D
RIP: RNA Immunoprecipitation
RISC: RNA-induced silencing complex
circRNAs: Circular RNAs
miRNAs: MicroRNAs
