# Supplementary material for: Circular RNA NEK6 contributes to the development of non-small-cell lung cancer by competitively binding with miR-382-5p to elevate BCAS2 expression at post-transcriptional level
Source: BMC Pulm Med. 2021 Oct 18;21:325. doi: 10.1186/s12890-021-01617-0 (PMC8524891; doi:10.1186/s12890-021-01617-0)
Supplement: Supplementary file 1 — Additional file 1: Fig. S1. Detailed information of circ_NEK6/miR-382-5p/BCAS2 axis in NSCLC. A. Pearson’s correlation analysis indicated the association of BCAS2 expression with the level of circ_NEK6 or miR-382-5p in 70 NSCLC tissues. B. Relationship between genes and NSCLC stages was shown. [file 12890_2021_1617_MOESM1_ESM.docx]

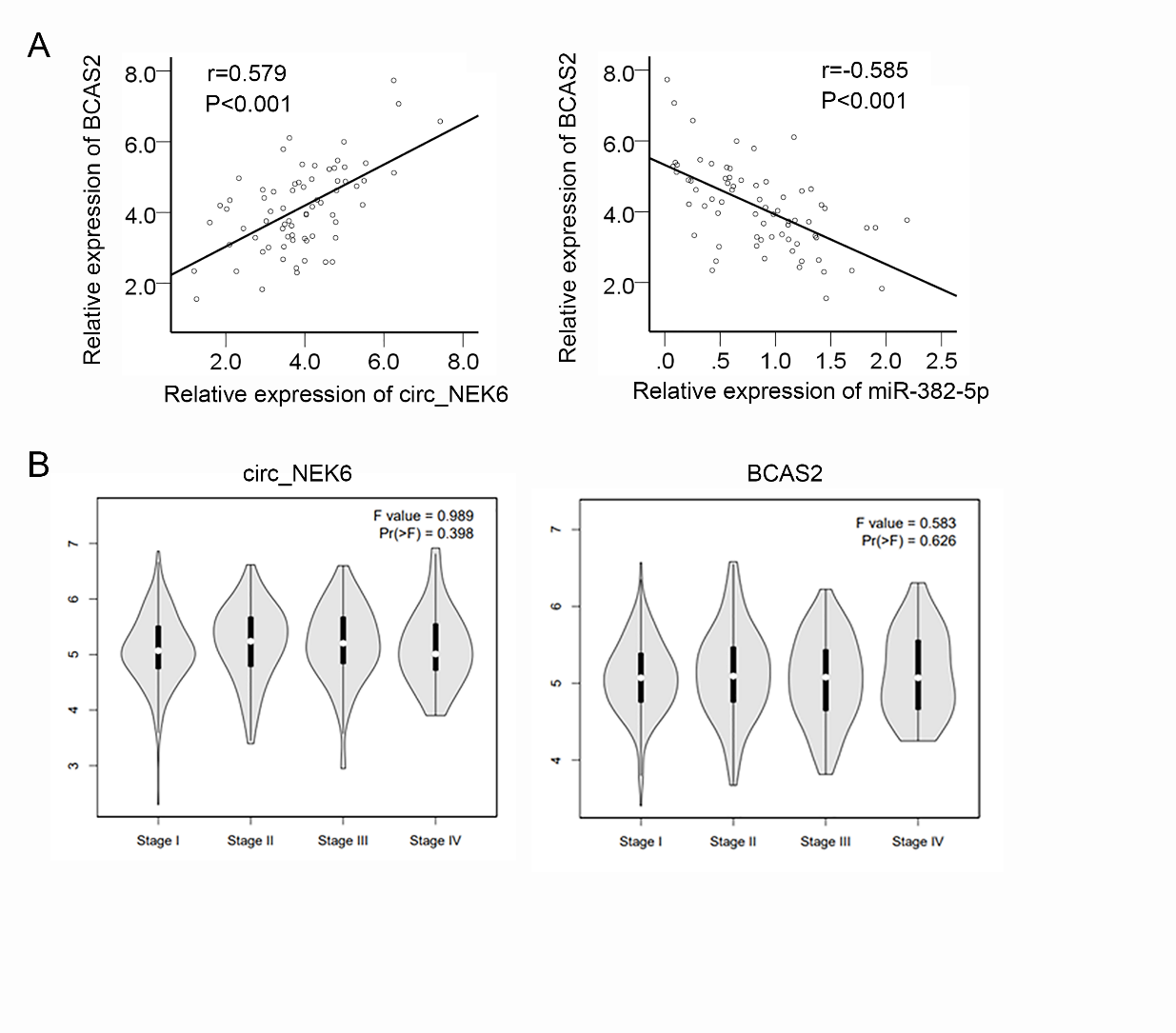


**Supplementary Figure 1 Detailed information of circ_NEK6/miR-382-5p/BCAS2 axis in NSCLC**

A. Pearson’s correlation analysis indicated the association of BCAS2 expression with the level of circ_NEK6 or miR-382-5p in 70 NSCLC tissues. B. Relationship between genes and NSCLC stages was shown.
