# Supplementary figures and images for: Circular RNA NEK6 contributes to the development of non-small-cell lung cancer by competitively binding with miR-382-5p to elevate BCAS2 expression at post-transcriptional level
Source: BMC Pulm Med. 2021 Oct 18;21:325. doi: 10.1186/s12890-021-01617-0 (PMC8524891; doi:10.1186/s12890-021-01617-0)

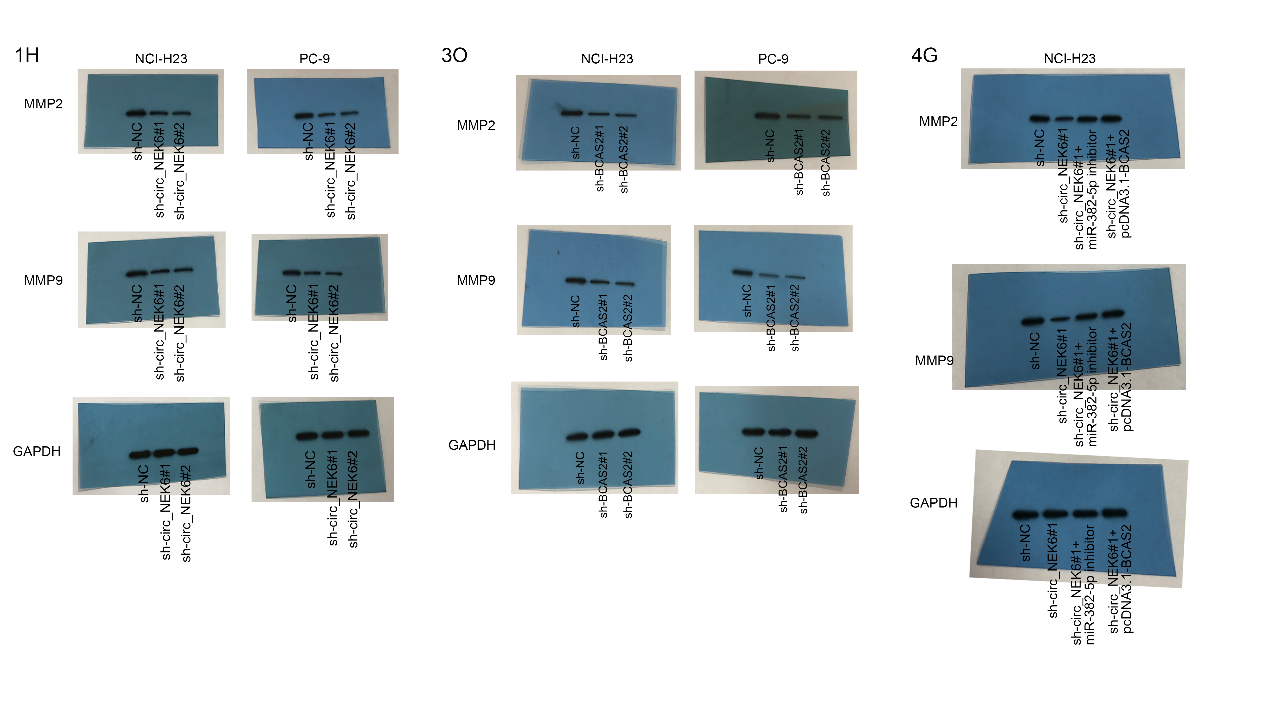


**Supplementary File 1 Original results of western blot assays in the laboratory**

Supplement: Supplementary file 2 — Additional file 2. Original results of western blot assays in the laboratory [file 12890_2021_1617_MOESM2_ESM.docx]

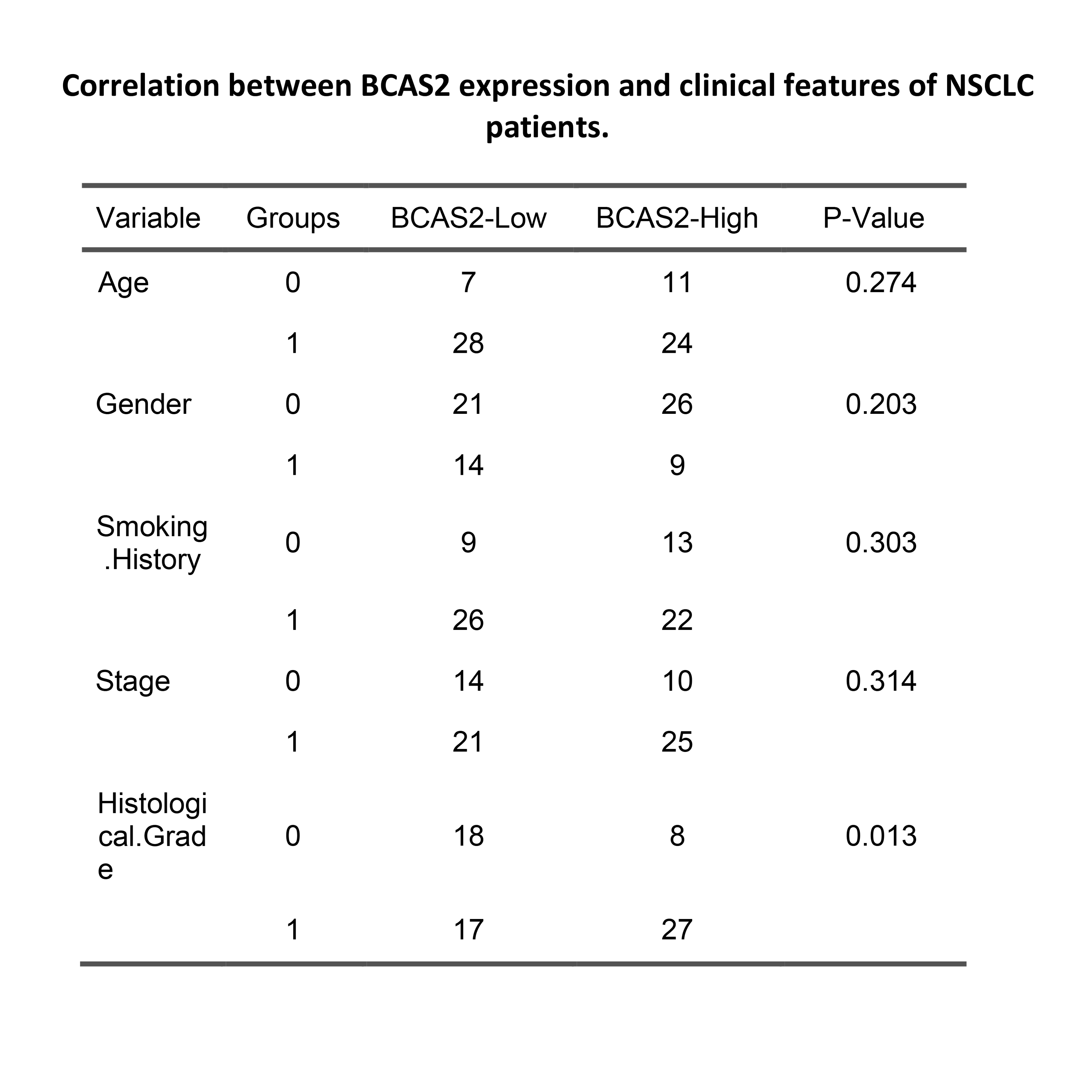

Supplement: Supplementary file 4 — Additional file 4: Table 2. Information on correlation between BCAS2 expression and clinical features of NSCLC patients [file 12890_2021_1617_MOESM4_ESM.tif]

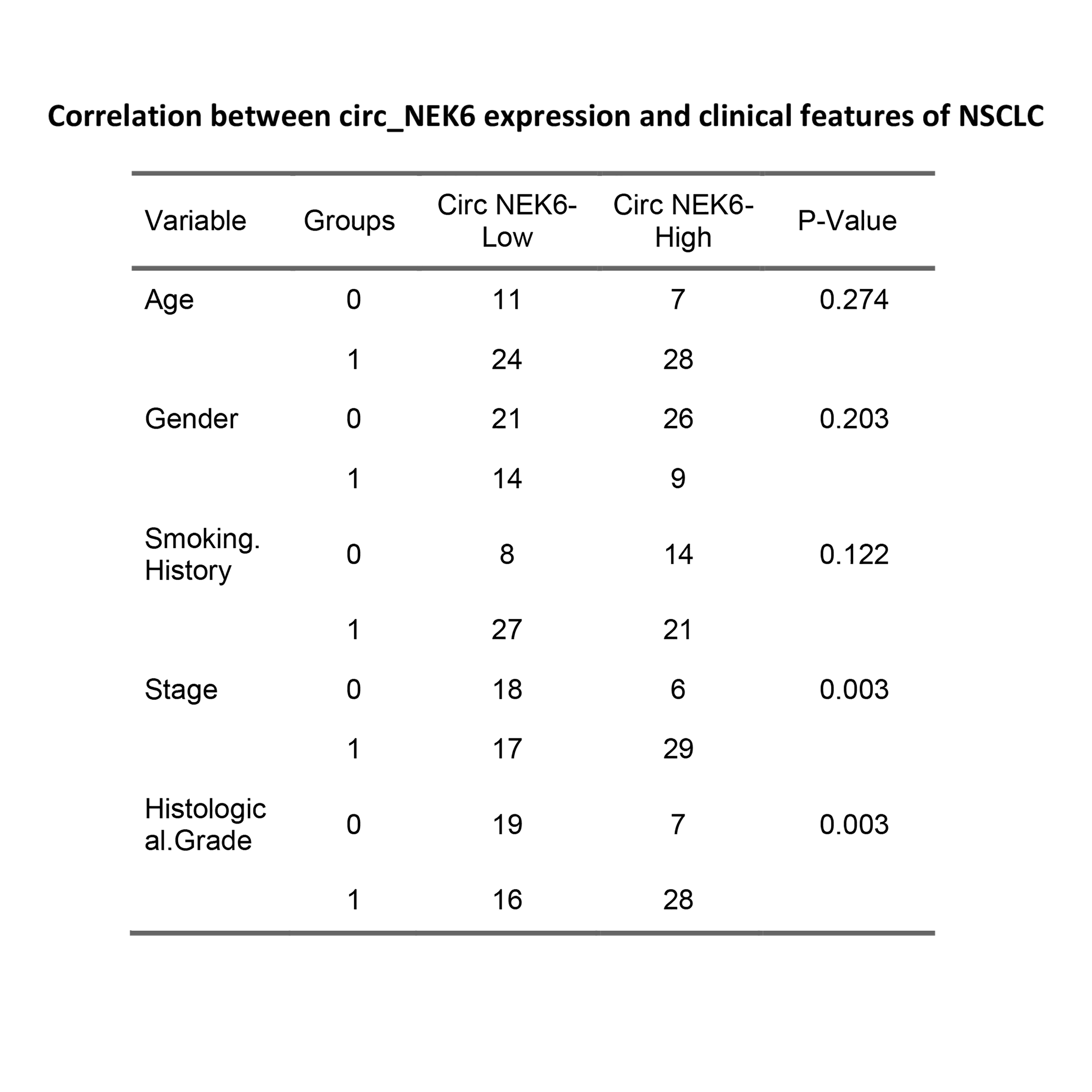

Supplement: Supplementary file 5 — Additional file 5: Table 3. Information on correlation between circ_NEK6 expression and clinical features of NSCLC patients [file 12890_2021_1617_MOESM5_ESM.tif]

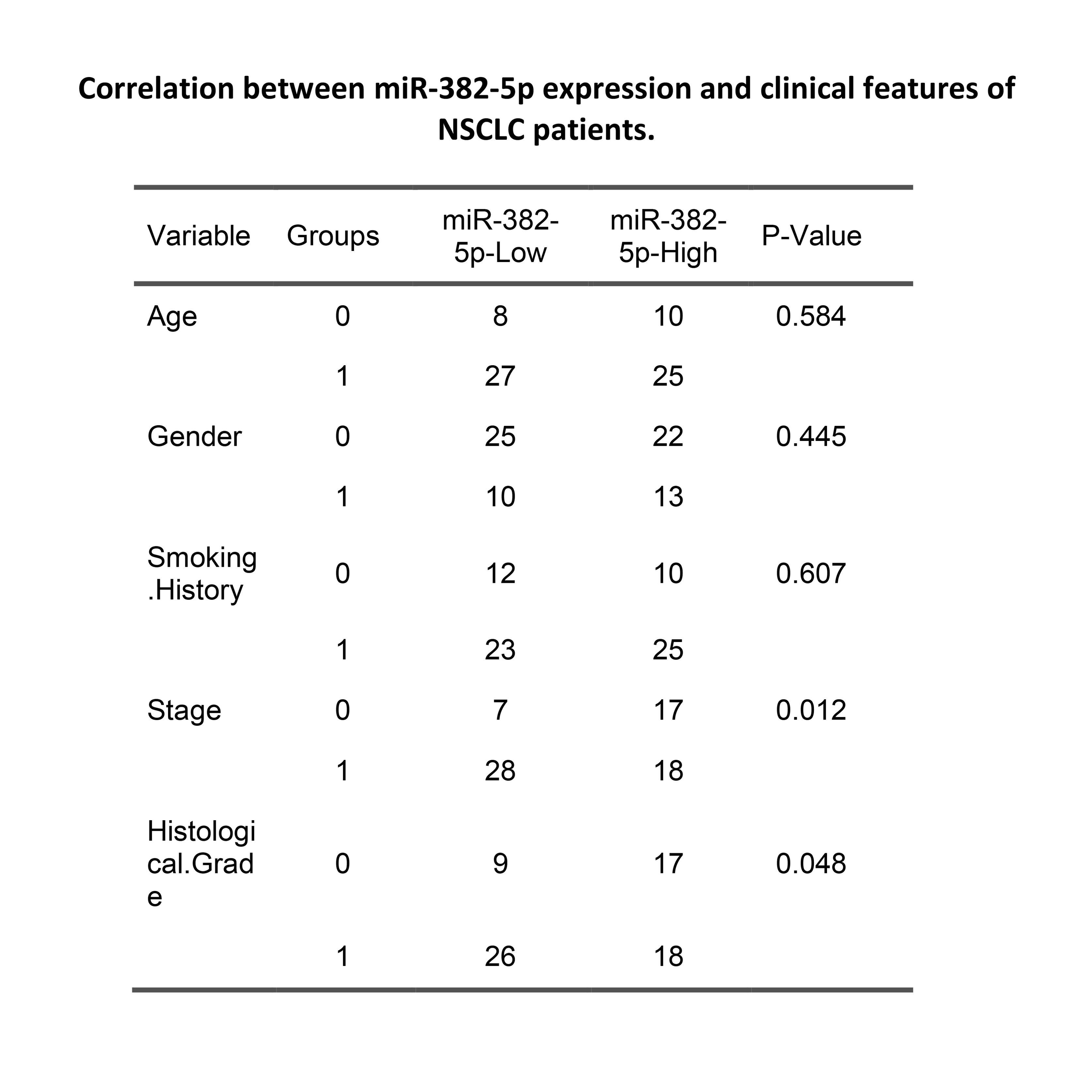

Supplement: Supplementary file 6 — Additional file 6: Table 4. Information on correlation between miR-382-5p expression and clinical features of NSCLC patients [file 12890_2021_1617_MOESM6_ESM.tif]
